# Association between apical periodontitis and psoriasis vulgaris: A cross‐sectional study

**DOI:** 10.1111/iej.14222

**Published:** 2025-03-19

**Authors:** Crystal Marruganti, Giulia Malvicini, Elisa Cinotti, Aurora Fuso, Emanuele Trovato, Pietro Rubegni, Simone Grandini, Carlo Gaeta

**Affiliations:** ^1^ Unit of Endodontics and Restorative Dentistry, Department of Medical Biotechnologies University of Siena Siena Italy; ^2^ Unit of Dermatology, Department of Medical, Surgical and Neurological Sciences University of Siena Siena Italy

**Keywords:** dental caries, periapical periodontitis, psoriasis

## Abstract

**Aim:**

To evaluate the prevalence of apical periodontitis (AP) and caries in subjects with psoriasis vulgaris.

**Methodology:**

In total, 152 patients with psoriasis vulgaris were included in the study. The severity and extent of psoriasis were assessed according to the Psoriasis Area Severity Index (PASI), the Body Surface Area (BSA) and the Physician's Global Assessment Scale (PGA). Periapical status was assessed through dental examination and periapical radiographs. Data regarding the Periapical Index (PAI), caries experience expressed as the Decayed, Missing, Filled Teeth Index (DMFT) and psoriasis medications were recorded. A predictive logistic regression model for the presence of AP and a linear regression model were then built to relate the severity and extent of AP to the type of drug therapy taken for psoriasis and to the severity and extent of the skin disease.

**Results:**

Subjects with severe/moderate psoriasis showed a significantly higher prevalence of AP (*p* = .002) and a higher PAI score (*p* = .0035) than subjects with mild psoriasis. No significant correlation was found between AP and caries experience (*p* = .76). The logistic regression model showed that moderate/severe psoriasis increased the odds of having AP [odds ratio (OR) = 1.30 ± 1.088, 1.55]. A negative linear relationship between biological drug intake and PAI score value was observed (coefficient = −.54; *p* = .04).

**Conclusions:**

The degree of severity of psoriasis is significantly associated with AP, suggesting that psoriasis may play a role in the pathogenesis of AP. However, no significant correlation was observed for caries experience. Furthermore, the immune‐modulating drugs taken by these patients did not seem to have different effects on the prevalence of AP.

## INTRODUCTION

Apical periodontitis (AP) is an oral inflammatory disease that occurs in the periradicular tissues as a result of a dynamic interaction between endodontic pathogens, their toxins and the host defence mechanism (Cotti & Schirru, 2022; Ye et al., 2023). Recent studies have demonstrated that AP has a significant systemic repercussion, but due to its often‐asymptomatic presentation, its global burden is often underestimated compared to other clinically visible oral conditions (Jakovljevic et al., 2020, 2021, 2023; Nagendrababu et al., 2020).

Psoriasis is a chronic relapsing inflammatory disease of autoimmune origin, with a multifactorial genetic predisposition (Trovato et al., 2023), currently regarded as a systemic disease affecting up to 4% of the population (Costa et al., 2021). Different forms of psoriasis have been described; however, psoriasis vulgaris is the most prevalent type (Oji & Luger, 2015). Typical clinical manifestations include sharply demarcated, scaly and erythematous plaques (Zhou & Yao, 2022). Despite its significant influence on quality of life, available treatments are often insufficient (Tokuyama & Mabuchi, 2020). Current treatments include topical therapies, phototherapy, systemic immune modulators and biologics, aiming to alleviate symptoms and improve quality of life (Lee & Kim, 2023). Many scoring systems have been developed to assess the severity of the disease; however, the most commonly used systems are the Psoriasis Area and Severity Index (PASI) and the Body Surface Area (BSA) (Looney et al., 2023; Marruganti, Romandini, et al., 2024).

Psoriasis has been reported to cause some mucosal lesions involving the oral cavity (Talaee et al., 2017). Over the past few years, it has been demonstrated that psoriasis is associated with oral health and that patients with psoriasis show a higher need for dental interventions (Olejnik et al., 2021). Furthermore, a recent study revealed that an efficient periodontal therapy improves the psoriasis condition in patients with periodontitis and psoriasis (Marruganti, Romandini, et al., 2024; Ucan Yarkac et al., 2020). Although there is strong evidence that links periodontitis with psoriasis, data on the association between psoriasis and periapical lesions is still scarce (Allihaibi et al., 2023). Furthermore, whilst the impact of immune‐modulating drugs on the prevalence of periapical lesions has been hypothesized for patients with autoimmune disorders (Allihaibi et al., 2023; Cotti et al., 2018; Piras et al., 2017) evidence specifically regarding psoriatic patients is still limited (Allihaibi et al., 2023).

Previous findings suggest that the oral–gut and skin–gut microbiota axes may support the hypothesis that AP could be correlated to the psoriasis‐related skin dysbiosis (Sinha et al., 2021). It was demonstrated that gut dysbiosis is linked to the development of various skin diseases, including psoriasis, making the gut–skin microbiota axis a key target to treat inflammatory skin disorders. Furthermore, the possible existence of an oral–skin microbiota axis, influenced by the gut composition, is supported by findings of altered salivary microbiota in individuals with psoriasis (Belstrøm, 2020). Additionally, several pro‐inflammatory cytokines such as TNF‐α, IL‐6, IL‐23 and IL‐17 are implicated in the onset and pathogenesis of both psoriasis and periapical lesions (Allihaibi et al., 2023; Furue et al., 2019; Ghoreschi et al., 2021; Lai & Dong, 2016; Nair, 2004; Wang & Jin, 2018).

A recent study investigated the presence of periapical lesions in patients with different autoimmune disorders, including psoriasis (Allihaibi et al., 2023). Amongst the included patients, 40 had psoriasis and 87% of them had AP, demonstrating a significant association between the two diseases. The authors of the study also evaluated the medications taken by the included subjects, focusing only on conventional systemic anti‐rheumatic drugs and biologics without focusing on other treatments such as phototherapy and topical medications (Trovato et al., 2023). Furthermore, the study did not evaluate psoriasis severity and did not consider which medications were associated with each autoimmune disease under study, highlighting the need for further research focusing on each singular disease. Additionally, the relatively small sample of patients with psoriasis underlines the necessity of additional studies with larger cohorts to ensure more robust and generalizable results.

The hypothesis that we would like to figure out is that the severity and extent of psoriasis are positively associated with a higher prevalence of AP and caries. Specifically, we hypothesize that more severe psoriasis correlates with increased periapical bone destruction and a higher caries experience, and that psoriasis medications may influence these oral health outcomes.

The null hypothesis of the present study is that there is no significant association between the severity of psoriasis and the prevalence or extent of AP and caries. It also posits that the treatment type does not significantly influence the prevalence of these oral conditions and their severity.

Thus, the aim of the present cross‐sectional study was to assess the presence and the extent of AP and caries in psoriasis vulgaris individuals stratified by disease severity. Additionally, the study examines whether medications associated with psoriasis treatment influence these oral health conditions.

## METHODOLOGY

### Study design

The present cross‐sectional study is reported according to the Preferred Reporting items for Observational studies in Endodontics (PROBE) guidelines (Nagendrababu et al., 2023). The research protocol was approved by the local Ethics Committee of Clinical Investigations of the Azienda Ospedaliero Universitaria Senese (protocol number: 18992) and received the registration number on Clinicaltrials.gov (NCT06436339). The study was conducted in accordance with the Declaration of Helsinki.

### Participants and setting

Participants were recruited from a specialty outpatient dermatology clinic (Unit of Dermatology, Azienda Ospedaliero‐Universitaria Senese, Siena, Italy) from February 2022 to November 2023. Inclusion criteria were as follows: (i) age between 18 and 70 years; (ii) diagnosis of psoriasis vulgaris (Nast et al., 2020); (iii) presence of at least 20 remaining teeth; (iv) ability and willingness to give informed consent. The exclusion criteria were as follows: (i) diagnosis of periodontitis (Tonetti et al., 2018); (ii) inability or unwillingness to give informed consent; (iii) periodontal treatment within the previous 6 months; (iv) ongoing topical or systemic immunosuppressive treatments or antibiotic therapy for other systemic diseases; (v) pregnancy or lactation; (vi) non‐endodontic lesions in the maxilla/mandible; and (vii) AP diagnosed on teeth with inadequate endodontic treatments and coronal restorations (Ng et al., 2011).

Subjects fulfilling the previously described criteria were enrolled from the outpatient department. Written informed consent was signed by all study participants prior to inclusion.

### Variables

#### Diagnosis of psoriasis

The diagnosis, severity and extent of the disease (mild, moderate or severe forms) were clinically established by one dermatologist (E.C.). Patients' personal details were collected, and their abdominal circumference was measured. Finally, the following dermatological variables were considered:

- PASI (Mrowietz et al., 2011). The body was divided into six regions: head, upper extremities, trunk and lower extremities. An ‘area score’ was established to describe the number of areas involved, ranging from 0 (no psoriasis) to 6 (total skin involvement). Each area was further assessed for severity based on thickness, scaliness, and redness, each rated from 0 to 4. Consequently, the total severity score for each region ranged from 0 to 12. The PASI score was calculated by multiplying the area score by the severity score, with a maximum potential score of 72 (6 × 12).
- BSA (Looney et al., 2023). An index that represents the proportion of the body affected by psoriasis, taking into account that one hand typically covers about 1% of the participant's total body surface area. It is also known as the ‘1% Hand Test’.
- PGA (Physician's Global Assessment Scale). A parameter that defines the severity of the pathology by correlating extension, intensity, and type of lesions (Langley & Ellis, 2004; Pascoe et al., 2015).

Furthermore, data regarding the patient's pharmacological therapy used to treat psoriasis were collected.

#### Oral examination

All the patients included were then directed to the Department of Endodontics and Restorative Dentistry of the University of Siena to receive extra‐ and intra‐oral examination.

The periapical status was assessed by palpation, percussion and thermal cold testing, along with panoramic x‐rays. Afterwards, teeth showing deep caries, deep restorations, lack of response to cold testing or painful response to biting and/or percussion or palpation were evaluated as potential cases of AP (American Association of Endodontists, 2013) and underwent additional periapical x‐rays using the long cone paralleling technique with a film holder (Duncan et al., 2023).

The following parameters were recorded:

- Number of Decayed, Missing and Filled teeth (DMFT) index;
- Teeth number;
- Periapical Index Score (PAI);
- Presence of AP;
- Periodontal status through periodontogram;
- Oral hygiene habits: frequency of brushing, type of toothbrush used, use of interproximal hygiene devices and mouthwashes;
- Lesions other than endodontic aetiology of the maxilla and mandible (Chauhan et al., 2019).

The PAI score (Ørstavik et al., 1986) was chosen for AP evaluation due to its widespread use and validated reliability in radiographic assessments and for its favourable connection with volumetric assessment of CBCT images (Rajasekhar et al., 2024). The PAI score was determined by visual examination of the periapical area on periapical radiographs, assigning a numerical value according to the extent and severity of the inflammation. The scoring system ranges from 0 to 5:

- Normal periapical structures;
- Minor alterations in bone structure;
- Changes in bone structure accompanied by slight mineral loss;
- Periodontitis with circumscribed bone and well‐defined halo of bone sclerosis;
- Severe periodontitis with extensive bone loss and a diffuse radiolucent appearance.

Scores 1 and 2 represent periapical health; Scores 3, 4 and 5 represent AP. The selection of the applied score was based on previously validated guidelines (Ørstavik et al., 1986). Two examiners (C.M. and A.F.) underwent a calibration process involving 100 standard radiographs that the index developers had already scored. Any discrepancies in their evaluations were resolved through discussion. This calibration procedure was repeated twice within a 2‐week interval to ensure consistency, and both inter‐ and intra‐observer agreements were quantified using kappa values. Following the calibration procedure, both examiners independently evaluated periapical radiographs of the teeth being studied under standardized conditions. The highest PAI score amongst the individual roots was considered for multirooted teeth. In cases of uncertainty, they reached an agreement and selected the higher scores. Importantly, during case assessment, the examiners were blinded to the identities and clinical conditions of the patients. Kappa statistics were used to assess intra‐ and inter‐observer agreement (Landis & Koch, 1977).

Furthermore, the quality of root canal treatment and coronal restoration of endodontically treated teeth was evaluated. Indeed, the quality of the root canal treatments and the coronal restorations was judged by the same examiners who assessed PAI scores, following the criteria described by Ng et al. (2011). Precisely, the quality of the previous treatment was considered satisfactory if a well‐compacted root filling extended to within 2 mm of the radiographic root apex (Ng et al., 2011). If the quality of either the root canal treatment or the coronal seal was not within the standard, the entire treatment was considered inadequate. Only those patients in whom AP was diagnosed on teeth with adequate endodontic treatments and coronal restorations were considered eligible for inclusion.

### Covariates

Subjects were interviewed regarding medical and family history, which included smoking habits, through a categorical assessment (yes/no/ex) and family history regarding diabetes, rheumatoid arthritis (RA), chronic inflammatory bowel diseases (IBDs), osteoporosis, cardiovascular diseases, chronic kidney disease and metabolic syndrome.

The values of the dermatological and endodontic variables were used for the prediction models. Moreover, self‐reported details [age, gender, body mass index (BMI), education, occupation and family history of periodontitis] were also considered. Eventually, data about medications taken for psoriasis was recorded from patient dermatological data and categorized as no therapy or topical/phototherapy, systemic medications (e.g. methotrexate, ciclosporin) and monoclonal antibodies (anti‐TNF‐alpha, anti‐IL‐17 and anti‐IL‐23).

### Sample size

The sample size calculation was based on the null hypothesis that the prevalence of AP in the present sample was the same as reported in a previous study (Allihaibi et al., 2023). It was assumed that the study cohort would have a 10% higher prevalence. With significance (*α*) set at .05 and statistical power (*β*) at .80, the calculated sample size was 110 subjects. Considering a non‐response rate of 30%, 152 participants were planned for inclusion, ensuring adequate statistical power for the study.

### Statistical analysis

Statistical analysis was conducted using ad hoc software (STATA BE 17.1, StataCorp, Texas, USA). Continuous variables were regarded as means and standard deviations (SD), whilst binomial or categorical variables were regarded as numbers of observations (*N*) and proportions (percentage %). Verification of the normal distribution of data was assessed using the Shapiro–Wilk test.

Continuous variables were compared using the Kruskal–Wallis test followed by Dunn's test, whilst binary and categorical variables were compared using the chi‐square test. Data regarding AP (number of periapical lesions and PAI score), caries experience (DMFT) and sociodemographic variables were compared according to the severity of psoriasis, expressed through PASI, categorized as mild when PASI was <5, moderate with PASI between 5 and 9 and severe with PASI >9, and according to the type of pharmacological therapy taken for psoriasis and divided into three categories: no therapy/topical therapy/phototherapy, traditional systemic therapy (cyclosporine, methotrexate, etc.) and biological drugs.

A logistic regression analysis was performed to identify predictive factors for the development of AP. A multivariable logistic regression model was conducted to evaluate the association between psoriasis (independent variable) and the occurrence of AP cases (dependent variable). The association between AP and psoriasis was expressed as crude and adjusted odds ratios (ORs). ORs were adjusted for age, gender, smoking, diabetes, AR, IBDs, osteoporosis and DMFT, which are parameters that could affect the AP phenotype and were selected according to external knowledge.

A chunk test was then performed (allsets command) to obtain the best possible regression model based on the highest value of area under the curve (AUC), the lowest value of Akaike information criterion (AIC) and Bayesian information criterion (BIC). AUC was used to assess the ability of the model to discriminate between outcomes. AIC and BIC were applied to assess the balance between model fit and complexity, with lower values indicating a simpler and well‐fitting model avoiding overfitting. This model aimed to establish whether the severity of psoriasis can predict the presence of AP. Other predictors (age, abdominal circumference, DMFT) were entered into the model.

A linear regression model was then built to correlate the severity and extent of AP with the type of pharmacological therapy taken and the severity of psoriasis. The best model will be chosen based on the lowest Mallows Cp value. The final regression model contains other covariates (abdominal circumference and DMFT). Statistical analysis was conducted setting the significance level at α = .05.

## RESULTS

### Patients' characteristics

**FIGURE 1:**
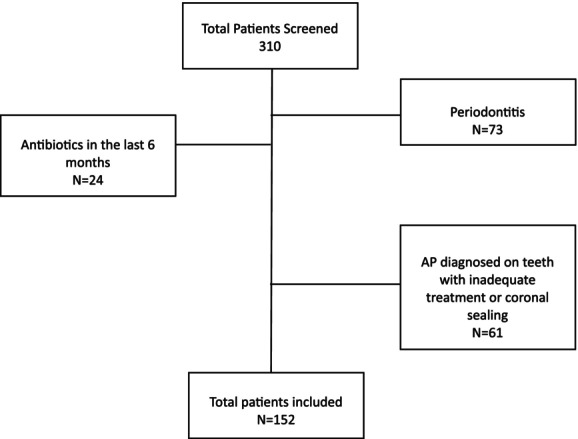
Flow chart for subject selection.

A total of 152 subjects were enrolled in the study (Figure 1). All individuals satisfying the eligibility criteria accepted to participate and were included in the analysis. Participants' characteristics are reported in Table 1. The intra‐examiner agreement for the PAI score resulted in kappa = 0.76 (95% CI: 0.69–0.79; *p* < .05) for the first examiner and kappa = 0.79 (95% CI: 0.71–0.84; *p* < .05) for the second examiner; inter‐examiner agreement resulted in substantial (kappa = 0.74, 95% CI: 0.69–0.81; *p* < .05). The mean age was 53 ± 16.38 years, with a proportion of 55.92% of males and 44.08% of females; the mean BMI was 26.56 ± 4.46; there were 52 smokers (34.21%), 16 patients with diabetes (10.53%), 29 with rheumatoid arthritis (19.08%) and 28 with cardiovascular disease (18.42%). The mean PaSI value was 7.97 ± 4.48 and the BSa value is 5.54 ± 4.36. Amongst subjects, 95 (62.5%) were taking medications for Psoriasis, with 14 of them having PASI ≥ 10 (9.21%).

**TABLE 1:** Socio‐demographic and anamnestic characteristics of all patients enrolled in the study.

Variable	Mean ± standard deviation number (%)
Age	53 ± 16.38
Gender	
Male	85 (55.92%)
Female	67 (44.08%)
Occupation	
Unemployed	21 (13.83%)
Employed	78 (51.32%)
Retired	53 (34.78%)
Education	
Primary school	43 (28.29%)
Secondary school	82 (53.95%)
University	27 (17.76%)
Smoking	
Non‐smoker	42 (27.63%)
Ex smoker	57 (37.50%)
Smoker	52 (34.21%)
Familiarity periodontal diseases	
No	95 (62.91%)
Yes	57 (37.50%)
Diabetes	
No	112 (73.68%)
Familiarity	24 (15.79%)
Yes	16 (10.53%)
RA	
No	112 (73.68%)
Familiarity	11 (7.24%)
Yes	29 (19.08%)
IBDs	
No	139 (91.45%)
Familiarity	3 (1.97%)
Yes	10 (6.58%)
Osteoporosis	
No	134 (88.16%)
Familiarity	8 (5.26%)
Yes	10 (6.58%)
Cardio‐vascular disease	
No	101 (66.45%)
Familiarity	23 (15.3%)
Yes	28 (18.42)
Chronic kidney disease	
No	150 (98.68%)
Yes	2 (1.32%)
Metabolic syndrome	
No	150 (98.68%)
Yes	2 (1.32%)
BMI	26.56 ± 4.46
Abdominal circumference	96.94 ± 17.59
PASI	2.97 ± 4.48
BSA	2.54 ± 4.36
PGA	1.13 ± 1.17
Phramacological therapy for psoriasis	
None/topical/phototherapy	57 (37.50%)
Systemic	20 (13.16%)
Biological antibodies	75 (49.34%)
Psoriasis severity	
Mild (PASI < 5)	115 (75.66%)
Moderate (PASI 5–9)	23 (15.13%)
Severe (PASI > 9)	14 (9.21%)
DMFT	9.3 ± 5.87
DT	0.89 ± 1.52
MT	2.98 ± 4.28
FT	5.36 ± 3.77
Number of periapical lesions	0.72 ± 1.01
PAI score	1.19 ± 1.70
AP	
No	79 (51.97%)
Yes	73 (48.03%)

Abbreviations: BMI, body mass index; DMFT, deacayed, missing, filled teeth; DT, decayed teeth; FT, filled teeth; IBDs, inflammatory bowel diseases; MT, missed teeth; *n*, number; RA, rehumatoid arthitis.

Regarding dental variables, 73 patients (48.03%) suffered from AP, with a mean PAI of 1.19 ± 1.70 and a mean DMFT of 9.3 ± 5.87.

Furthermore, patient characteristics were compared across three severity groups and three therapy groups (Tables 2 and 3). Results indicated comparability amongst the groups, with no significant differences observed in patients' age, gender, occupation, education, disease and family history, smoking status, BMI and DMFT. Significant differences were observed between the three severity groups in relation to AP, the number of periapical lesions and PAI. In contrast, no significant difference emerged between the three therapy groups in relation to these variables.

**TABLE 2:** Variables compared according to the severity of psoriasis.

Mean ± standard deviation number (%)				
Variable	Mild	Moderate	Severe	*p*‐value
	*n* = 115	*n* = 23	*n* = 14	
Age	53.7 ± 7.07	48.78 ± 13.73	56.83 ± 13.81	.21
Gender				
Male	65 (56.52%)	9 (39.13%)	11 (78.57%)	.07
Female	50 (43.48%)	14 (60.87%)	3 (21.43%)	
Occupation				
Unemployed	14 (12.17%)	5 (21.74%)	2 (14.29%)	.56
Employed	58 (50.43%)	13 (56.52%)	7 (50%)	
Retired	43 (37.39%)	5 (21.74%)	5 (35.71%)	
Education				
Primary school	34 (29.57%)	6 (26.09%)	3 (21.43%)	.52
Secondary school	63 (54.78%)	13 (56.52%)	6 (42.86%)	
University	18 (15.65%)	4 (17.39%)	5 (35.71%)	
Smoking				
Non‐smoker	36 (31.30%)	4 (17.39%)	2 (14.29%)	.15
Ex smoker	8 (34.78%)	8 (34.78%)	6 (42.86%)	
Smoker	11 (47.83%)	11 (47.83%)	6 (42.86%)	
Diabetes				
No	86 (74.78%)	16 (69.57%)	10 (7.43%)	.82
Familiarity	16 (13.91%)	5 (21.74%)	3 (21.43%)	
Yes	13 (11.39%)	2 (8.70%)	1 (7.14%)	
RA				
No	85 (73.91%)	16 (69.57%)	11 (78.57%)	.32
Familiarity	6 (5.22%)	4 (17.39%)	1 (7.14%)	
Yes	24 (20.87%)	3 (13.04%)	2 (14.29%)	
IBDs				
No	106 (92.17%)	21 (91.30%)	12 (85.71%)	.06
Familiarity	1 (0.87%)	2 (8.7%)	0	
Yes	8 (6.96%)	0	2 (14.29%)	
Osteoporosis				
No	101 (87.83%)	20 (86.96%)	13 (92.86%)	.14
Familiarity	4 (3.48%)	3 (13.04%)	1 (7.14%)	
Yes	10 (8.70%)	0	0	
Cardiovascular disease				
No	78 (67.83%)	14 (60.87%)	9 (64.29%)	.23
Familiarity	14 (12.17%)	7 (30.43%)	2 (14.29%)	
Yes	23 (20.00%)	2 (8.70%)	3 (21.43%)	
Chronic kidney diseases				
No	113 (98.26%)	23 (100%)	14 (100%)	1
Yes	2 (1.74%)	0	0	
Metabolic syndrome				
No	113 (98.26%)	23 (100%)	14 (100%)	1
Yes	2 (1.74%)	0	0	
Pharmacological therapy for psoriasis				
None/topical/phototherapy	39 (33.91%)	8 (34.78%)	10 (71.43%)	.00*
Systemic	11 (9.57%)	6 (26.09%)	3 (21.43%)	
Biological drugs	65 (56.52%)	9 (39.13%)	1 (7.14%)	
BMI	26.22 ± 4.19	27. 41 ± 5.72	27 ± 4.20	.35
DMFT	9.36 ± 6.15	8.56 ± 4.16	10 ± 13.81	.76
DT	0.91 ± 1.64	0.91 ± 1.20	0.71 ± 0.82	.97
MT	3.15 ± 4.53	2.3 ± 2.96	2.78 ± 4.09	.91
FT	5.23 ± 3.83	5.34 ± 3.29	6.5 ± 4.07	.43
Number of periapical lesions	0.53 ± 0.74	1.04 ± 1.10	1.78 ± 1.84	.00*
PAI score	0.91 ± 1.44	1.69 ± 1.91	2.71 ± 2.39	.00*
Periapical lesions				
No	69 (60%)	7 (30.43%)	3 (21.43%)	.00*
Yes	49 (40%)	16 (69.57%)	11 (78.57%)	

Abbreviations: AP, apical periodontitis; BMI, body mass index; DMFT, decayed, missing, filled teeth; DT, decayed teeth; FT, filled teeth; IBDs, inflammatory bowel diseases; MT, missed teeth; *n*, number; PAI, periapical Index; RA, rheumatoid arthritis.

* *p* < .05.

**TABLE 3:** Variables compared according to the drug therapy taken for psoriasis.

Mean ± standard deviation number (%)				
Variable	None /topical/phototherapy	Systemic drugs	Biological drugs	*p*‐value
	*n* = 57	*n* = 20	*n* = 75	
Age	52.49 ± 18.97	55.63 ± 15.77	53.19 ± 14.46	.58
Gender				
Male	29 (50.88%)	11 (55%)	45 (60%)	.60
Female	28 (49.12%)	9 (45%)	30 (40%)	
Occupation				
Unemployed	10 (17.54%)	1 (5%)	10 (13.33%)	.13
Employed	24 (42.11%)	9 (45%)	45 (60%)	
Retired	23 (40.35%)	10 (18.87%)	20 (26.67%)	
Education				
Primary school	14 (24.56%)	9 (25%)	24 (32%)	.24
Secondary school	29 (50.88%)	10 (50%)	43 (57.33%)	
University	14 (24.56%)	5 (25%)	8 (10.67%)	
Smoking				
Non‐smoker	17 (29.82%)	5 (25%)	20 (26.67%)	.25
Ex smoker	26 (45.61%)	6 (30%)	25 (33.33%)	
Smoker	14 (24.56%)	9 (45%)	30 (40%)	
Diabetes				
No	19 (68.42%)	16 (80%)	57 (76%)	.65
Familiarity	12 (21.05%)	3 (15%)	9 (12%)	
Yes	6 (10.53%)	1 (5%)	9 (12%)	
RA				
No	45 (78.95%)	18 (90%)	49 (65.33%)	.11
Familiarity	5 (8.77%)	0	6 (8%)	
Yes	7 (12.28%)	2 (10%)	20 (26.67%)	
IBDs				
No	55 (96.49%)	18 (90%)	66 (88%)	.31
Familiarity	1 (1.75%)	0	2 (2.67%)	
Yes	1 (1.75%)	2 (10%)	7 (9.33%)	
Osteoporosis				
No	50 (87.72%)	17 (85%)	67 (89.33%)	.85
Familiarity	4 (7.02%)	1 (5%)	3 (4%)	
Yes	3 (5.26%9	2 (10%)	5 (6.67%)	
Cardiovascular disease				
No	36 (63.16%)	15 (75%)	50 (66.67%)	.85
Familiarity	10 (17.54%)	3 (15%)	10 (13.33%)	
Yes	11 (19.30%)	2 (10%)	15 (20%)	
Chronic kidney disease				
No	56 (98.35%)	20 (100%)	74 (98.67%)	1
Yes	1 (1.75%)	0	1 (1.33%)	
Metabolic syndrome				
No	57 (100%)	0	73 (97.33%)	.62
Yes	0	0	2 (2.67%)	
AP				
No	30 (52.63%)	10 (50%)	39 (52%)	1
Yes	27 (47.37%)	10 (50%)	36 (48%)	

Abbreviations: AP, apical periodontitis; BMI, body mass index; IBDs, inflammatory bowel diseases; *n*, number; PAI, periapical Index; RA, rheumatoid arthritis.

### Regression model

A logistic regression model was built to identify potential predictors for the occurrence of AP (Table 4). A chunk test was also conducted to identify the best model using AUC (discriminative ability, AUC = 0.72) and the lowest AIC (195.2) and BIC (210.3) values, ensuring model fit and simplicity. Some key factors such as the severity of psoriasis, age, abdominal circumference and DMFT were included in the model as predictors. The analysis revealed statistical significance (*p* < .05) with the model's ability to explain the variance in the response variable estimated at 31% (pseudo *R* ^2^). Specifically, the regression model highlights that the presence of a moderate/severe degree of psoriasis significantly raises the likelihood of AP by approximately 1.3 times (OR = 1.30 [1.088–1.55]; *p* = .00).

**TABLE 4:** Final logistic regression model analysing predictive factors related to apical periodontitis (AP).

Best model (AIC = 195.2; AUC = 0.72; BIC = 210.3)						
LR χ^2^	Prob > χ^2^	Pseudo *R* ^2^				
22.6	.00	.3087				
**AP**	**OR**	**SE**	***z***	***p*‐value**	**95% CI**	
					**Lowest value**	**Highest value**
Psoriasis Moderate/Severe	1.3	.11	2.89	.00*	1.08	1.55
Age	1.02	.01	1.60	.10	.99	1.04
Abdominal circumference	1.01	.01	1.34	.18	.99	1.03
DMFT	1.05	.02	1.57	.11	.98	1.12
*_cons*	.03	.04	−3.00	.00	.00	.31

Abbreviation: DMFT, decayed, missing, filled teeth.

* *p* < .05.

Regarding the other predictors, age (OR = 1.02 [0.99–1.04]; *p* = .10), abdominal circumference (OR = 1.01 [0.99–1.03]; *p* = .18) and DMFT (OR = 1.05 [0.98–1.12]; *p* = .11) did not demonstrate a statistically significant association with AP in the regression model.

The aim of the linear regression model (Table 5) was to establish a relationship between the severity and the extent of periapical bone destruction (PAI), and the severity of psoriasis and the type of pharmacological therapy administered. Notably, psoriasis severity did not emerge as a covariate in the final best model. The covariates retained in the final model include biologic drug intake, abdominal circumference and DMFT. The model shows that compared to alternative therapies such as traditional systemic drugs, topical therapies, phototherapy or no therapy, the use of biological drugs results in a significant reduction in the PAI score by 0.54 (*p* = .04). Additionally, an increase of one point in the DMFT (continuous variable) corresponds to a rise in the PAI score by approximately 0.44, a finding that also bears statistical significance (*p* = .03). Overall, the model exhibits statistical significance (*p* < .05) with a 31% interpretability rate (*R* ^2^).

**TABLE 5:** Results of the linear regression model.

Best model (Mallows's Cp = 1.72)						
PAI score	Coefficient	ES	*t*	*p*‐value	95% CI	
					Lowest highest	
Biological drugs	−.54	.27	−1.99	.04*	−1.07	−.00
Abdominal Circumference	.01	.00	1.74	.08	−.00	.02
DMFT	.44	.02	2.09	.03*	.02	.59
*_cons*	−.27	.79	−.34	.73	−1.83	1.29

			Source	Analysis of variance		Mean squares
				Sum of squares	gl	
*R* ^2^	.31		Model	34.55	3	11.51
ADJ *R* ^2^ adjusted	.27		Residual	402.64	146	2.75
Root MSE	1.66		Total	437.17	149	2.93
			*F* = 4,17; *p* = .0072			

Abbreviations: DMFT, decayed, missing, filled teeth; gl, degrees of freedom; PAI, periapical index; SE, standard error of the mean.

* *p* < .05.

## DISCUSSION

The results of the present study show that patients suffering from psoriasis have an increased prevalence of AP compared to the general population. Logistic regression analysis revealed a significant correlation between moderate/severe psoriasis and AP occurrence, indicating a 1.3‐fold increase in likelihood. Furthermore, the use of biological drugs is significantly associated with lower PAI scores.

The aim of the current cross‐sectional study was to investigate whether psoriasis could be identified as a predictor for AP and whether its treatment could influence the extent and severity of periapical bone destruction. A large body of evidence associates psoriasis with periodontitis (Chen et al., 2012; Costa et al., 2021; Majchrzycka et al., 2022; Marruganti, Gaeta, et al., 2024; Sarac et al., 2017; Sezer et al., 2016; Ucan Yarkac et al., 2020); conversely, data on the potential consequences on periradicular tissues of psoriasis are still scarce. Despite their different aetiologies and pathogenesis, AP and periodontitis are both polymicrobial infections sharing a similar microbiota, often composed of Gram‐negative anaerobic bacteria (Sundqvist, 1992), and characterized by an increased systemic level of cytokines (Cotti & Mercuro, 2015). Given these similarities, one might suppose that AP might also be associated with psoriasis. Indeed, a recent study demonstrated that patients with autoimmune disorders, including psoriasis, whether treated or not with biologic medications, showed a higher prevalence of AP than those in the control group (Allihaibi et al., 2023). Our findings slightly differ from those of this previous report in which 87% of patients with psoriasis also had AP. In contrast, in the present study, almost 50% of psoriasis patients were found to have AP. Discrepancies might be explained by differences in the design of the study, sample size, but also in patients' characteristics (Allihaibi et al., 2023).

From the findings of the present study, it is possible to suppose that psoriasis may play a role in the pathogenesis of AP. This might be due to the role of pro‐inflammatory cytokines (i.e. TNF‐α, IL‐6, IL‐23 and IL‐17) that can be found in the onset and pathogenesis of both AP and Psoriasis (Allihaibi et al., 2023; Furue et al., 2019; Ghoreschi et al., 2021; Lai & Dong, 2016; Nair, 2004; Wang & Jin, 2018). Another possible explanation relies on the role of the oral–gut and skin–gut microbiota axes (Sinha et al., 2021). Indeed, it was observed that gut dysbiosis is linked to the development of various skin disorders, including psoriasis. Additionally, evidence exists regarding the potential presence of an oral‐skin microbiota axis influenced by the gut microbiota, as individuals with psoriasis have shown changes in the salivary microbiota (Belstrøm, 2020).

Results from the logistic regression analysis revealed a significant association: individuals with severe/moderate psoriasis showed a 1.3‐fold higher probability of presenting AP (*p*‐value = .004). This suggests that psoriasis severity may serve as a predictor for AP occurrence.

Our findings did not show any significant difference between the three therapy groups in relation to AP prevalence and PAI scores. This means that patients who are taking biological drugs have the same probability of developing AP as those who are taking alternative or no therapies. These results differ from previous studies which reported that patients who are taking biologic medications had a higher prevalence of AP (Allihaibi et al., 2023; Ideo et al., 2022; Piras et al., 2017). Differences in AP prevalence observed in our study compared to prior research may be attributed to variations in disease type, patient populations, study designs and radiographic evaluation methods. Our study specifically focused on patients with psoriasis, whilst prior studies often focused on other autoimmune diseases with different inflammatory profiles, without accounting for the medications taken by specific disease groups (Allihaibi et al., 2023). Furthermore, our study categorized patients with psoriasis in different psoriasis severity levels, whereas other studies did not make such distinctions. Additionally, variations in comorbidities amongst patients could further contribute to the observed differences in AP prevalence and outcomes. Indeed, patients taking biological drugs are likely to be in more advanced stages of autoimmune diseases, as these drugs are typically administered to patients who did not respond to other therapies (Allihaibi et al., 2023).

However, despite no significant difference being highlighted in terms of therapy, in the group of patients taking biological drugs, almost half (48%) had AP. It was demonstrated that biologic drugs may not have positive effects on the prevalence of AP in the absence of treatment, but they could influence the results of endodontic treatment when used in association with it (Cotti et al., 2018; Piras et al., 2017). A recent study demonstrated that the treatment of AP in patients taking biologic drugs was associated with faster healing compared to the control group (Cotti et al., 2018).

The linear regression model highlights that biological drugs compared to alternative therapies such as traditional systemic drugs, topical therapies, phototherapy or no therapy result in a significant decrease in the PAI score by 0.54 (*p* = .04). This finding suggests that whilst biologic drugs may not change the probability of developing AP, their ability to modulate excessive cytokine activities may reduce the destruction of periapical tissues (Cotti et al., 2018). The lack of significance for the PAI score across different therapy groups in the inferential statistics is due to its reliance on unadjusted group comparisons, which do not consider confounding factors. In contrast, the linear regression model adjusts for covariates, isolating the effect of biological drugs, revealing a significant reduction in PAI scores.

In the present study cohort, the mean DMFT value was 9.3 ± 5.87. This aligns with findings from a previous perspective study analysing the oral health of 201 patients affected by psoriasis, where the mean DMFT was 12.26 ± 7.08 (Woeste et al., 2019). Notably, this value did not significantly differ from the control group (mean DMFT: 12.13 ± 7.02; *p* = .86). Our findings similarly indicated no discernible disparity in DMFT across severity groups.

The linear regression model revealed a significant association between increasing DMFT (continuous variable) and PAI score (*p* = .03), suggesting a predisposition to greater AP severity and extent with higher DMFT values, as expected. It is therefore plausible that the worse overall dental health of patients suffering from psoriasis results in higher numbers of teeth affected by AP, as confirmed in Allilhaibi's study (Allihaibi et al., 2023).

In the present study, around 34% of the patients are smokers (Table 1). Smoking could influence the AP phenotype; however, instead of excluding it, its impact was assessed through statistical adjustments and subgroup analyses (Gaeta et al., 2025; Malvicini et al., 2024). ORs were indeed adjusted for age, gender, smoking, diabetes, RA, IBDs, osteoporosis, and DMFT. The best model was selected based on the highest value of AUC and the lowest values of AIC and BIC information criteria, identifying age and abdominal circumferences as significant predictors. Adjusting for smoking minimized its confounding effects and ensured a rigorous analysis.

The current study presents some limitations. First, cross‐sectional studies do not allow for establishing causation and exclude reverse causality. For this purpose, prospective interventional studies would be necessary. Secondly, the absence of microbiological or immunological analysis prevents us from confirming possible theories regarding the relationship between the two diseases. Thirdly, even though the major confounding factors were addressed in the multiple models, the risk of residual confounding should be considered. Fourthly, the sample size calculation focused on the primary outcome (AP); therefore, it cannot be excluded that some estimates related to secondary outcomes may have large confidence intervals due to a decrease in statistical power. Additionally, although periapical x‐rays are commonly used for AP diagnosis, their ability to detect changes in periapical bone is limited, potentially leading to underdiagnosis (Cotti & Schirru, 2022). Cone‐beam computed tomography (CBCT) stands as the current gold standard, ensuring early diagnosis of periapical lesions (Cotti & Schirru, 2022)ll periapical lesions (Cotti & Schirru, 2022). Moreover, this study was conducted in a specialty dermatology clinic, which may limit the generalizability of the study to a broader population, as patients often present with more severe cases. Geographic, cultural and healthcare access differences could also influence the findings. Ultimately, another important shortcoming relies on the operator‐dependent component of the DMFT index. Therefore, the findings should be interpreted cautiously, recognizing the limitations inherent in the study design.

The results from the present study highlight the importance of integrating oral health into the care of psoriasis patients, who may be at higher risk of developing AP. Understanding the interaction between the host's predisposition to psoriasis and the potential effect of the therapy on the development of AP may be useful to design new treatment strategies for AP and a patient‐centred approach. Improving collaboration between dermatologists and dentists can help to address the shared inflammatory mechanisms underlying these conditions. Future investigations should aim to establish a causal relationship, integrating patient‐reported outcomes and consider conducting multicentric studies to validate findings across different populations and healthcare settings.

## CONCLUSIONS

The findings of the present cross‐sectional study indicate a significant association between psoriasis severity and AP, suggesting that psoriasis may contribute to the occurrence of AP or vice versa. In contrast, no significant relationship was observed between caries experience and AP prevalence. Whilst immune‐modulating drugs did not appear to affect the prevalence of AP differently, the DMFT index may be regarded as a significant predictor of AP severity. These results underscore the need for further research to clarify the causal mechanisms linking psoriasis and AP, as well as to explore the potential impact of psoriasis severity on endodontic treatment outcomes.

## AUTHOR CONTRIBUTIONS

**Crystal Marruganti:** data curation, formal analysis, investigation, methodology; **Giulia Malvicini:** data curation, writing—original draft and editing; **Elisa Cinotti:** conceptualization, methodology, data curation; **Aurora Fuso:** data curation, investigation; **Emanuele Trovato:** supervision, writing—review and editing; **Pietro Rubegni:** data curation, investigation; **Simone Grandini:** data curation, methodology; **Carlo Gaeta:** supervision, investigation, methodology.

## FUNDING INFORMATION

No external funding, apart from the support of the authors' institution, was available for this study.

## CONFLICT OF INTEREST STATEMENT

The authors deny any conflict of interest related to this study.

## ETHICS STATEMENT

Approved by the University Hospital of Siena Ethics Committee (Siena, Italy), Area Vasta Toscana Sud Est, protocol number 18993/2021. The present observational study is reported according to the Preferred Reporting items for OBservational Studies in Endodontics (PROBE) guidelines.

## INFORMED CONSENT

All enrolled patients were informed about the study protocol and were asked to read and sign the informed consent. The present study was conducted according to the Declaration of Helsinki.

## Data Availability

The data sets used and/or analysed during the current study are available from the corresponding author upon reasonable request.
